# Association between eHealth literacy and health outcomes in German athletes using the GR-eHEALS questionnaire: a validation and outcome study

**DOI:** 10.1186/s13102-024-00902-9

**Published:** 2024-05-24

**Authors:** Sheila Geiger, Anna Julia Esser, Matthias Marsall, Thomas Muehlbauer, Eva-Maria Skoda, Martin Teufel, Alexander Bäuerle

**Affiliations:** 1 Clinic for Psychosomatic Medicine and Psychotherapy, LVR-University Hospital Essen, University of Duisburg-Essen, Essen, Germany; 2 Centre for Translational Neuro- and Behavioral Sciences (C-TNBS), University of Duisburg- Essen, 45147 Essen, Germany; 3 Institute for Patient Safety (IfPS), University Hospital Bonn, Bonn, Germany; 4 Division of Movement and Training Sciences, Biomechanics of Sport, University of Duisburg- Essen, Essen, Germany

**Keywords:** Health-related outcomes, Sports, Digital survey, Internet, Discriminant and convergent validity, Factorial structure

## Abstract

**Background:**

Athletes face various sports-related stressors, which may increase their risk for physical and mental health symptoms. With the internet as an important source of (health) information, it is important for athletes to have eHealth literacy, i.e. the ability to access, understand and use electronic health information and services. However, it is presently uncertain whether eHealth literacy of athletes is linked to better health outcomes such as reduced injury frequency and behaviours like decreased substance abuse.

**Methods:**

A cross-sectional study was conducted with *N* = 373 German athletes (229 females) from different types of sport (e.g., ball sports and water sports) who were included in the statistical analyses. The survey included medical, socio-demographic, eHealth- and sports-related data as well as the eHealth Literacy Scale (GR-eHEALS) questionnaire, which measures eHealth literacy. Confirmatory factor analyses and correlational analyses were performed to determine the convergent and discriminant (compared to the 8-item Impulsive Behavior–8 Scale) validity of the GR-eHEALS and to assess the relation between eHealth literacy scores and health outcomes.

**Results:**

The more frequently athletes had sustained minor or moderate injuries in the past, the higher the level of eHealth literacy they reported. Furthermore, consumption frequency of painkillers (*r* = .18, *p* = .002), sedatives (*r* = .12, *p* = .040), and cannabis (*r* = .29, *p* = .000) was significantly correlated with eHealth literacy scores. The confirmatory factor analysis of the GR-eHEALS showed an acceptable model fit with a 2-factor solution (information seeking and information appraisal). The GR-eHEALS showed good discriminant (*r* = − .09, *p* = .21) and convergent validity (digital confidence; *r* = .28, *p* < .001).

**Conclusion:**

The GR-eHEALS is a valid instrument to assess eHealth literacy within the cohort of German athletes. Potential dangers of dealing with injury and psychological strain without reaching out for professional help should be considered.

**Supplementary Information:**

The online version contains supplementary material available at 10.1186/s13102-024-00902-9.

## Background

Athletes participating in sports at the highest levels of performance and competition [1] are confronted with a range of risk factors and physical stress due to intensive training. The prevailing belief is that a substantial training load serves as a crucial stimulus for enhancing athletic performance [2]. However, an imbalance between training intensity and the appropriate recovery periods may lead to various impairments, such as overtraining syndrome or relative energy deficiency [3, 4]. Engaging in professional sports subjects the body to considerable effort and strain, frequently leading to various sports-related injuries [5]. Further challenges include performance and competition pressure, financial issues, a lack of social support [6], and the potential for involuntary retirement due to injuries [7]. Moreover, athletes may be particularly susceptible to mental health symptoms and disorders as a result of the unique stressors associated with their sport [6]. Research has also indicated that young people, particularly athletes, are less likely to seek professional help for mental health issues [8, 9]. Instead, athletes often turn to substances, such as painkillers, sedatives, and cannabis, to boost their performance, reduce stress, regulate their emotions, and alleviate pain [10, 11].

Therefore, it is important that athletes are able to access, understand, and use health information in a way that promotes and maintains health on a long-term basis, meaning that they raise their level of health literacy [11]. The concept of literacy has undergone many changes over time, from the basic skills of reading and writing to a multidimensional concept of data-processing, including cognitive abilities and social aspects [12]. Low literacy in itself is often associated with poor health outcomes [13]. The concept of health literacy has two distinct roots - in clinical care and in public health - and is therefore considered a clinical risk or a personal asset [11]. From the clinical perspective, poor literacy skills are seen as a potential risk factor that needs to be managed in the process of providing clinical care. Following the definition of Ratzan and Parker [14], the US Institute of Medicine report defines health literacy as: “the degree to which individuals have the capacity to obtain, process, and understand basic health information and services needed to make appropriate health decisions” (p. 1). This conceptualisation suggests that health literacy relies partly on knowledge and can be enhanced through educational interventions [11]. Regarding public health, health literacy is viewed as a valuable resource to cultivate, resulting from health education that enhances empowerment in making health-related decisions [11]. This understanding of health literacy originates from educational research on literacy, as well as from theories of adult learning and health promotion [10, 15]. The World Health Organization (WHO) also considers health literacy to be an evolving concept [16] and, following Nutbeam's definition, describes it as the ability of individuals to ”gain access to, understand and use information in ways which promote and maintain good health” (p. 10; [17]), which is subtly different to the definition of the Institute of Medicine.

Literature indicates a substantial correlation between health literacy and various critical health outcomes. Specifically, individuals with low health literacy tend to experience higher rates of hospitalizations, increased reliance on emergency care, lower adherence to medication, limited understanding of health-related information, suboptimal self-management behaviour, and overall poorer health status [18–20]. Regarding athletes, critical health outcomes include among others the number and severity of injuries and substance use.

In the present day, information concerning health-related subjects is not exclusively disseminated by professionals. The internet provides an overwhelming array of opportunities to obtain (health) knowledge [21]. The use of electronic health (eHealth) technologies has become increasingly prevalent in recent years, and eHealth literacy, i.e. the ability to access, understand, and utilize health-related information and services through electronic means, has become an important aspect of healthcare [22]. Multiple studies have demonstrated that higher eHealth literacy is linked to positive outcomes in both the general population and clinical groups, i.e., improved health knowledge [23, 24], enhanced health information seeking [25, 26], increased health intention [27], greater engagement in preventive health behaviour [24, 28], and improved adherence to healthcare recommendations [29]. Additionally, there is a positive association between eHealth literacy and beneficial health behaviours in non-athletes, such as maintaining a healthy diet and regular exercise [30, 31]. eHealth literacy can be particularly advantageous for athletes due to their tendency to hold less positive attitudes toward seeking help for mental health issues as compared to non-athletes [32]. Moreover, their extensive travel for national and international competitions and limited time spent at home further emphasizes the potential benefits of eHealth literacy, as eHealth technologies can be used regardless of location or time [33]. It is currently unclear how far eHealth literacy has advanced among athletes and how it may affect their health.

We examined the eHealth Literacy Scale (GR-eHEALS; 34), which is based on the eHEALS by Norman and Skinner [35], as a means of assessing eHealth literacy among German athletes. The eHEALS is to date the most widely used instrument to assess eHealth literacy [35, 36] and was developed as a measure designed for broad use in supporting consumer eHealth in public health and clinical care [22]. The GR-eHEALS is a valid and reliable assessment instrument for measuring eHealth literacy in the German language [34]. The GR-eHEALS comprises the two cognitive processes of information seeking and information appraisal [34]. In this regard, we devised two objectives to achieve with this study: firstly, to test the factorial structure of the GR-eHEALS in German athletes and assess its construct validity by examining both convergent and discriminant validity; and secondly, to explore the associations between eHealth literacy and health-related outcomes (i.e. substance use and injuries).

## Methods

### Study design and participants

This digital survey was conducted as a cross-sectional study, adhering to the approval guidelines of the Ethics Committee of the Faculty of Medicine at the University of Duisburg-Essen (19-8947-BO). The results reported in this study are part of a larger survey-based study, of which partial results have already been published [37]. Prior to the survey, each participant provided electronic informed consent. Participation was both anonymous and voluntary, without any form of reimbursement. We utilized the Unipark software (Tivian XI GmbH) that was distributed through social media, sports clubs (involving athletes competing in regional and nationwide tournaments), and sports associations (both regional and nationwide) from December 2021 to December 2022. The eligibility criteria included being an adult (≥ 18 years old), possessing a good command of the German language, having internet access, and being an athlete. An athlete was defined as someone who prioritizes sports in their life, strives for athletic excellence, and participates in professional or Olympic competitions [38–41]. The assessment of whether the participants were athletes was based on self-report. The survey received responses from a total of 651 participants. After excluding participants who do not meet the aforementioned criteria for athletes (*n* = 166) or reported an age below 18 years or above 90 years (*n* = 112), the total sample was reduced to *N* = 373.

### Measures

This study collected sociodemographic data from participants through self-report measures, including information on their sex, age, marital status, education level and financial situation. Furthermore, sports-related data (i.e., type of sports and whether they do individual or team sports) was assessed. eHealth literacy of participants was assessed using the GR-eHEALS [34], which is based on the eHEALS by Norman and Skinner [35]. The GR-eHEALS consists of eight items that are rated on a 5-point Likert scale (1 = do not agree at all; 5 = fully agree). The questionnaire measures the two cognitive processes of eHealth literacy with the two subscales information seeking and information appraisal. The first subscale focuses on searching for information on the Internet (e.g. "I know where I can find helpful health information on the Internet"). The second subscale describes the step of evaluating the information found (e.g. "I can distinguish between trustworthy and dubious websites with health information").

To test the convergent validity of the GR-eHEALS, established scales measuring digital confidence [42, 43] were administered. Furthermore, the length of daily internet use for personal and professional purposes was evaluated using a single self-developed item rated on a 5-point Likert scale (1 = not at all; 5 = more than 5 hours). In addition, three items each were inquired after internet anxiety and digital overload [42, 44], and they were rated on a 5-point Likert scale (1 = totally disagree; 5 = totally agree). These measures were expected to correlate significantly with the GR-eHEALS, as per Campbell and Fiske [45] guidelines. To evaluate the discriminant validity of the GR-eHEALS, we used the 8-item Impulsive Behavior–8 Scale [46] to measure impulsivity as a personal trait that was expected to be independent of eHealth literacy, as impulsive people tend to act rashly [47] without thinking carefully or reflecting their consequences [48], whereas eHealth literacy describes the cognitive ability of accessing, understanding and using information in a health-promoting way [11]. The items of the scale were also rated on a 5-point Likert scale.

Additionally, participants provided medical data through self-report measures. It was assessed how often the following substances were consumed on a 5-point Likert scale (1 = never, 5 = daily): Cannabis, nicotine, sedatives prescribed by physicians (e.g. benzodiazepines), painkillers prescribed by physicians (e.g. tramadol), sedatives not prescribed by physicians / over-the-counter sedatives, painkillers not prescribed by physicians / over-the-counter painkillers (e.g. ibuprofen, diclofenac). Moreover, number and severity of injuries was assessed. For this purpose, athletes indicated on a 5-point Likert scale (1 = not at all, 5 = more than 20 times a year) how often they had suffered minor, moderate and severe injuries within the last year and how often surgery had been necessary. The complete questionnaire in English can be found in the supplementary material.

### Statistical analyses

Statistical analyses were conducted with R version 4.2.2.2 and R Studio 2023.06.1 + 524. A confirmatory factor analysis (CFA) was performed in order to affirm the factor structure of the GR-eHEALS scale in the present sample. Results were interpreted according to Hu and Bentler [49] assuming the comparative fit index (CFI) and Tucker Lewis index (TLI) of at least 0.95 and root mean square error of approximation (RMSEA) and standardized root mean square residual (SRMR) of below 0.06 and 0.08, respectively [49]. As the GR-eHEALS consists of items on ordinal scale, a robust likelihood estimator (WLSMV; [50]) was chosen to avoid biases in the model. Internal consistencies (reliability) of the convergent and discriminant validity scales, the GR-eHEALS and its two subscales were examined. Subsequently, two-tailed Pearson correlations were conducted between the validity scales, the outcome measurements, and sociodemographic variables with the GR-eHEALS. Sex differences on GR-eHEALS were assessed by a two-tailed independent *t*-Test. Results were considered as significant with *p* = .05. Incomplete data was deleted list wise.

## Results

### Sample characteristics

Participants were *M* = 23.48 (*SD* = 6.13) years old, half was younger than *Mdn* = 22.00. The sample consisted of 229 (61.39%) female, 140 (37.53%) male and 4 (1.07%) diverse participants. On average participants had a body-mass-index (BMI) of *M* = 22.89 kg/m^2^ (*SD* = 2.42 kg/m^2^). Participants averaged a daily internet use of 1–3 hours in a private context (*M* = 3.23, *SD* = 0.73) as well as 1–3 hours in a professional one (*M* = 2.90, *SD* = 1.02). Further sample characteristics can be taken from Table 1.

**Table 1:** Description of the study sample

Variable		Complete sample		Complete GR-eHEALS sample	
		*n*	%	*n*	%
**Educational level**					
University degree		87	23.32	65	23.05
Highschool diploma		212	56.84	164	58.16
Vocational training		16	4.29	14	4.96
Secondary school certificate		24	6.43	16	5.32
Still in school education		24	6.43	15	5.43
No degree		3	0.80	2	0.71
Other		7	1.88	5	1.77
**Marital status**					
Married		24	6.45	19	68.09
In relationship		80	21.51	64	22.70
Single		260	69.89	192	68.09
Divorced/Separated		2	0.54	2	0.71
Widowed		3	0.81	0	0
Other		3	0.81	3	1.06
**Sports**					
Individual sports		257	68.63	194	68.79
Team sports		175	46.91	136	48.23
Ball sports		121	32.44	93	32.98
Martial arts		27	7.24	19	6.74
Weightlifting		19	5.09	16	5.67
Athletics		33	8.85	27	9.57
Cycling		1	0.27	1	0.35
Equitation		26	6.97	17	6.03
Gymnastics		17	4.56	11	3.90
Dancing		11	2.95	5	1.77
Water sports		105	28.15	84	29.79
Winter sports		2	0.54	2	0.71
Trend sport and other		11	2.94	7	2.48

Sample characteristics in absolute numbers (*n*) and in percent (*%*) for the whole sample (left column; *N* = 373) and for the sample with complete data on all GR-eHEALS variables (right column; *N* = 282), respectively

### Internal consistency of the scales

Convergent validity scales showed good and excellent internal consistency. Digital confidence (*M* = 4.12, *SD* = 0.74) showed excellent internal consistency of Cronbach's α = 0.90. Good internal consistency was achieved by digital overload (*M* = 2.72, *SD* = 0.97) with Cronbach's α = 0.73 and by internet anxiety (*M* = 1.84, *SD* = 0.79) with Cronbach's α = 0.79. Impulsivity (*M* = 2.67, *SD* = 0.56) as discriminant validation scale reached good internal consistency of Cronbach's α = 0.71.

### Construct validation of the GR-eHEALS in athletes

To confirm the factorial structure of the GR-eHEALS in our sample, we performed a confirmatory factor analysis (CFA, see Table 2). The CFA showed a good model fit with the two-factor solution (information seeking and information appraisal) previously reported [34]. Excellent internal consistency was reached by the total GR-eHEALS score and good internal consistency by its subscales information appraisal and information seeking. Table 3 shows the items and statistics of the GR-eHEALS.

**Table 2:** Results of CFA to confirm the factorial structure of the GR-eHEALS

Model	Chi² (*df*)	*p*	CI	CFI	TLI	RMSEA	SRMR
1	71.409 (19)	< .001	0.075, 0.124	0.917	0.878	0.099	0.043

*Chi²* Chi²-coefficient, *df *degrees of freedom, *p* p-value, *CI* 90% confidence interval (lower, upper), *CFI* comparative fit index, *TLI* Tucker Lewis index, *RMSEA* root mean square error of approximation, *SRMR* standardized root mean square residual

**Table 3:** Item statistics of the GR-eHEALS (*N* = 282)

	Item	*α*	*M*	*SD*	Skew	Response distribution %				
						1	2	3	4	5
	**eHealth Literacy total score**	0.93	3.65	0.78	-0.49	-	-	-	-	-
	**Information seeking**	0.89	3.58	0.89	-0.43	-	-	-	-	-
1	I know how to find websites with helpful health information.	0.91	3.71	0.96	-0.85	2.78	10.42	17.01	52.43	17.36
2	I know how to use the Internet to get answers to my health questions.	0.92	3.77	0.93	-0.79	1.39	11.11	15.28	53.12	19.10
3	I know what health information sites are available on the Internet.	0.91	3.36	1.13	-0.39	4.86	23.61	15.28	42.71	13..54
4	I know where to find helpful health information on the Internet.	0.92	3.47	1.08	-0.49	2.78	22.92	12.50	47.92	13.89
	**Information appraisal**	0.86	3.71	0.79	-0.63	-	-	-	-	-
5	I know how to use health information from the Internet in a way that helps me.	0.91	3.71	0.95	-0.87	2.78	9.72	17.71	53.12	16.67
6	I am able to critically evaluate websites with health information.	0.92	3.85	0.92	-1.04	2.78	6.25	15.62	53.82	21.53
7	I can distinguish between trustworthy and dubious health information websites.	0.91	3.75	0.95	-0.98	2.43	11.46	11.81	57.64	16.67
8	I feel confident in using information from the Internet to make decisions regarding my health.	0.92	3.44	1.02	-0.36	1.77	21.28	19.86	43.97	13.12

*M *mean, *SD* standard deviation, *α* internal consistency, standardized Cronbach’s *α, *when item dropped. The items of the GR-eEHALS used in the survey were in German and have been translated for publication purposes

### Correlation analysis

#### Demographic data and eHealth literacy scores

Neither participants’ financial situation (*M* = 7.01, *SD* = 2.20, *r* = − .04, *p* = .499), nor their age (*r* = 0.11, *p* = .601) was associated with their eHealth literacy scores. Female participants (*M* = 3.62, *SD* = 0.80) did not score differently on eHealth literacy compared to male participants (*M* = 3.71, *SD* = 0.76; *t* (246.23) = 0.95, *p* = 0.346).

### Correlations with the convergent and discriminant validity scales

Half of the participants scored above *Mdn* = 4.00 on digital confidence, above *Mdn* = 2.67 on digital overload as well as above *Mdn* = 1.67 on internet anxiety. The higher individuals rated their digital confidence (*M* = 4.12, *SD* = 0.74), the higher eHealth literacy they reported (*r* = .28, *p* < .001). However, there was no correlation between digital overload (*M* = 2.73, *SD* = 0.98) and eHealth literacy (*r* = − .02, *p* = .79) nor between internet anxiety (*M* = 1.85, *SD* = 0.81) and eHealth literacy (*r* = .04, *p* = .49). Discriminant validity was confirmed as impulsivity (*M* = 2.67, *SD* = 0.56) was not associated with eHealth literacy (*r* = − .09, *p* = .21).

### Correlations with outcome measurements

Participants’ descriptions of their injury severity and frequency can be seen in Table 4. The more frequently participants were injured with minor severity (*r* = 0.14, *p* = .019) and moderate severity (*r* = .21, *p* < .001), the higher they scored on the eHealth literacy scale, whereas there was no significant relation between eHealth literacy with severe injuries (*r* = 0.09, *p* = .135). The frequency of surgical intervention in the past year did not correlate with eHealth literacy scores either (*r* = 0.10, *p* = .087).

Participants’ consumption frequency of cannabis, nicotine, general sales list tranquilizers, and painkillers was positively associated with their indicated eHealth literacy. Descriptive statistics and other correlations between eHealth literacy scores and substance consumption can be seen in Table 5.

**Table 4:** Descriptive statistics of injury severity and frequency per year (*N* = 373)

	Minor		Moderate		Severe		Surgery	
***M*** **±** ***SD***	2.27 ± 0.94		1.56 ± 0.65		1.23 ± 0.50		1.16 ± 0.43	
**Missings**	3.75%		4.29%		4.02%		4.83%	
**Frequency**	***n***	**%**	***n***	**%**	***n***	**%**	***n***	**%**
**0**	80	22.28	185	51.82	283	79.05	303	85.35
**1–2**	140	38.40	145	40.62	72	20.11	50	14.08
**3–5**	108	30.08	26	7.28	1	0.28	0	0.00
**6–20**	25	6.96	0	0.00	0	0.00	1	0.28
**> 20**	6	1.67	1	0.28	2	0.56	1	0.28

*Missings* percentage of missing data in each response category, *frequency* injury frequency per year, minor, moderate, severe, *surgery* severity of the injury, *M ± SD* mean and standard deviation

**Table 5:** Correlation between eHealth literacy scores with the frequency of substance use

	*M* ± *SD*	eHealth literacy	
		*r*	*p*
Cannabis	1.33 ± 0.92	0.29	.000**
Nicotine	1.52 ± 1.15	0.22	.000**
Sedatives prescribed by physicians (e.g. benzodiazepines)	1.12 ± 0.55	-0.06	.293
Painkillers prescribed by physicians (e.g. tramadol)	1.35 ± 0.84	0.08	.161
Sedatives not prescribed by physicians/over-the-counter sedatives	1.46 ± 1.11	0.12	.040*
Painkillers not prescribed by physicians/ over-the-counter painkillers (e.g. ibuprofen, diclofenac)	3.02 ± 1.31	0.18	.002**

*M ± SD* mean ± standard deviation, *r* correlation coefficient, * significant with p<.05, ** significant with p<.01.

## Discussion

The study aimed to assess both the factorial structure and construct validity of the GR-eHEALS, while also investigating the relationships between eHealth literacy among athletes and their health outcomes. The following major novel findings emerged from this study: a) The confirmatory factor analysis of the GR-eHEALS demonstrated an acceptable model fit, revealing a 2-factor solution comprising information seeking and information appraisal. Moreover the GR-eHEALS showed good discriminant and convergent validity. b) Athletes’ scores on eHealth literacy were positively and significantly correlated with the frequency of minor and moderate severe injuries. Athletes’ consumption frequency of cannabis, free sales list tranquilizer and painkillers were positively associated with eHealth literacy.

### Methodological examination

In accordance with our hypothesis and in accordance with previous literature [34, 42], the results indicated good discriminant and convergent validity of the GR-eHEALS and an appropriate model fit with a two-factor solution as suggested by Marsall, Engelmann [34]. Thus, the GR-eHEALS is a valuable instrument to assess eHealth literacy in an athlete’s cohort. In line with our expectations, eHealth literacy was associated with digital confidence, showing good convergent validity. As described in previous research, digital confidence is a key factor to find and understand appropriate health information online [51]. With regard to discriminant validity, the expected lack of or weak correlation between GR-eHEALS and patient impulsivity was confirmed by the analyses conducted.

Contrary to existing literature there was no correlation between eHealth literacy and digital overload, nor between eHealth literacy and internet anxiety [34, 42]. This could be explained by a floor effect [52]: As the present sample spent 1–3 hours per day on the internet, both privately and professionally, they were likely to be highly skilled in handling digital requirements, which might reduce internet anxiety as well as digital overload. Besides, the present sample is relatively young. Given that young individuals have grown up with the internet [53], they might have developed more adaptive strategies for managing the increasing array of digital demands and offerings, rather than experiencing a sense of being overwhelmed or anxious [54]. This interpretation is also supported by the high digital confidence in the present sample. Consequently, the scale used in the present study could not possibly distinguish between the low scores on internet anxiety and digital overload due to a presumed floor effect.

### Minor and moderate injuries correlate with eHealth literacy

In line with our hypothesis and consistent with previous literature [55–57], the sample indicated to have minor or moderate injuries once or twice per year. Contrary to our expectations, our sample did not show reduced injury frequencies with higher eHealth literacy. Instead, there was an association between minor and moderate injuries and higher eHealth literacy.

This may suggest that athletes handle their injuries mostly on their own as long as the severity of the injury allows it, which might be in accordance with the concept of eHealth literacy. The non-significant relation between eHealth literacy scores and the frequency of severe injuries supports this interpretation, as it suggests that severe injuries demand professional help immediately and do not allow self-help through research online anymore.

Our results could be further explained by the barriers athletes face when seeking for psychological or medical help [58]. Due to their strict training schedule, athletes might not find the time to make an appointment with physicians [59] or they might perceive injuries and pain as a default in their daily training and competition [60]. Thus, athletes might feel the need to handle pain without anyone knowing. The internet might offer an anonymous solution to this problem, deducible from the shown tendency to predominantly develop eHealth literacy when affected by less severe injuries.

### Association between substance use and eHealth literacy

Contrary to our hypotheses and a previous study examining health literacy and substance use in young people [61], we found a positive association between eHealth literacy and the consumption of cannabis, free sales painkillers and tranquilizers, but not with medically prescribed painkillers and sedatives. These findings might be explained by the desire to cope with pain or stress on one's own. Painkillers or cannabis can help to reduce pain, to prevent the development of pain memory and could beneficially influence inflammation [62, 63]. Thus, taking painkillers or cannabis might be in accordance with the concept of eHealth literacy. Free sales tranquilizers and cannabis are also an effective way to regulate emotional tension in the short term [64]. The use of substances as self-medication, regeneration support or performance improvement among athletes has been discussed in previous research [65].

Thus, on the one hand eHealth literacy seems to represent a helpful resource for athletes to cope with injuries and psychological strain on their own. On the other hand, previous research has shown that regular cannabis use has long-term harmful consequences, which contradicts the concept of eHealth literacy [66, 67]. Compulsive use of cannabis, e.g. to reduce stress, enhances the risk to develop an addiction memory [68]. Possibly, due to stigma and missing disclosure in competitive sports, athletes might engage in compulsive behaviours like substance use to regulate their stress [57].

###  Rethinking the results in the working context of competitive sports

High eHealth literacy usually indicates awareness for long-term negative health outcomes of substance use or training while on pain medication [18]. Therefore, the association between eHealth literacy and the use of painkillers, sedatives and cannabis consumption cannot be fully explained by the beneficial short-term outcomes and might be better understood by considering beliefs and assumptions common in competitive sports (e.g., “It was really, really drilled into us basically that you know if you were still getting your periods you probably weren’t training hard enough”) [69]. Some athletes reported on choosing to suppress pain in order to continue participating in their training program, which could provoke more severe injuries [60]. Despite potential knowledge about the risks of their behaviour, it might be more important and perhaps even more approved to momentarily function in their sports rather than to fully recover. Athletes’ attitudes supporting this interpretation have been described in qualitative studies [69, 70]. Thus, the high demands and barriers for help seeking in competitive sports might affect priorities in athletes’ decision making, which could lead to neglect of physical and mental health.

Moreover, the results could be ascribed to the high athletic demands athletes face. To reach full athletic potential while staying healthy potentially requires individually tailored health behaviours and this sport-specific adaption may require expert knowledge. Health behaviours resulting from research online may not fully meet the athlete’s needs. Hence, health impairments like injuries may occur despite high eHealth literacy. This underlines the necessity to offer awareness programs, prevention and help options for this vulnerable group, in order to avoid long-term health issues. Instead of exclusively applying performance-enhancing behaviour, the promotion of health enhancing behaviour could play a decisive role in competitive sports. The IOC (International Olympic Committee) has become aware of this topic and has begun to promote help seeking in athletes [71].

The results of this study should be interpreted under the caveat of certain limitations. As the study design was cross-sectional, causal conclusions could not be derived from the data. In addition, eHealth literacy was assessed through self-assessment only, which may not provide an accurate representation of skills and competences. A more accurate assessment method would involve comparing self-assessment with actual behaviour, but no such tool exists currently. Additionally, the assessment of whether the participants were athletes is also based on self-report, which means that athletes who did not actually meet the criteria may also have taken part. Furthermore, the survey was conducted online, which may have resulted in a sample that is more representative of individuals who are comfortable using the internet and digital devices and may not accurately reflect the views of those who are less familiar with the internet. This possibility of selection bias should be considered, however, it should be noted that internet use in Germany reached 91% of the population in 2021 and continues to increase [72]. As data was collected (i.e., December 2021 to December 2022) during the fading COVID-19 pandemic, mental health issues and help seeking behaviour might have been confounded [73–75]. Moreover, the present study only surveyed the frequency, but not the functionality of cannabis consumption or criteria of addiction, which should be considered in the interpretation regarding short- and long-term health outcomes.

## Conclusions

This study confirmed discriminant and convergent validity as well as the two-factor structure of the GR-eHEALS in a sample of athletes. Within the cohort of German athletes, the GR-eHEALS emerged as a valid instrument for assessing eHealth literacy. Athletes who had experienced minor or moderate injuries more frequently reported higher eHealth literacy. Additionally, the frequency of consuming painkillers, sedatives, and cannabis was positively correlated with eHealth literacy scores. Contrary to the general population, in competitive sports the association between eHealth literacy and positive health outcomes is inconsistent. Athletes were discussed to develop eHealth literacy in response to impaired health statuses. In the context of competitive sports, eHealth information might rather function to improve athletic performance, instead of engaging in health behaviours that prevent harmful health outcomes. Furthermore, high athletic demands might also require high specificity and expertise in terms of health information. Hence, athletes may not profit enough from health information online. These discoveries may suggest that additionally to developing eHealth literacy, implementing health professional teams in the field of competitive sports and a greater focus on health-promoting behaviours over performance-enhancing behaviours might be a promising step towards healthier athletes.

### Supplementary Information

Supplementary Material 1.

Supplementary Material 2.

## Data Availability

The dataset supporting the conclusions of this article is available in the DuEPublico2 repository, https://doi.org/10.17185/duepublico/81405.

## Abbreviations

CFA: confirmatory factor analysis
CFI: comparative fit index
Chi²: Chi²-coefficient
CI: confidence interval
df: degrees of freedom
ehealth: Electronic health
eHEALS: eHealth Literacy Scale
GR-eHEALS: german eHealth Literacy Scale
IOC: International Olympic Committee
M: mean
Mdn: median
M±SD: mean and standard deviation
n: sample
p: p-value
r: correlation coefficient
RMSEA: root mean square error of approximation
SD: standard deviation
SRMR: standardized root mean square residual
t: t-value
TLI: Tucker Lewis index
WLSMV: weighted least squares with mean and variance adjusted estimate
α: internal consistency, standardized Cronbach’s α
%: percentage
