# Supplementary material for: Association between eHealth literacy and health outcomes in German athletes using the GR-eHEALS questionnaire: a validation and outcome study
Source: BMC Sports Sci Med Rehabil. 2024 May 24;16:117. doi: 10.1186/s13102-024-00902-9 (PMC11127337; doi:10.1186/s13102-024-00902-9)
Supplement: Supplementary file 1 — Supplementary Material 1. [file 13102_2024_902_MOESM1_ESM.docx]

**Supplementary Material**

**Association Between eHealth Literacy and Health Outcomes in German Athletes using the GR-eHEALS Questionnaire: A Validation and Outcome Study**

Sheila Geiger^1,2*^, Anna Julia Esser^1,2*^, Matthias Marsall^3^, Thomas Muehlbauer^4^, Eva-Maria Skoda^1,2^, Martin Teufel^1,2^, Alexander Bäuerle^1,2^

The following elements were used in the study. The original study was in German and was translated into English for publication purposes.

**Are you currently an elite athlete according to the above criteria?**

- Yes
- No

**Do you play individual or team sports?**

**(multiple answers possible)**

- Individual sports
- Team sport

**Which sport do you practise at elite level?**

We have used broad generic terms and listed example sports below. Please select a category that suits your sport.

- BALL SPORT (badminton, baseball, basketball, (beach) volleyball, football, handball, hockey, tennis, table tennis, water polo ...)
- MATERIAL ARTS (boxing, fencing, judo, karate, wrestling, taekwando...)
- WEIGHTLIFTING
- ATHLETICS (running, multi-discipline event, jumping, throwing …)
- CYCLING (track cycling, BMX, mountain biking, road racing ...)
- EQUITATION (dressage, show jumping, eventing …)
- SPORTS SHOOTING (crossbow, bow, firearm)
- GYMNASTICS (floor gymnastics, apparatus gymnastics, rhythmic gymnastics, trampolining, vaulting ...)
- DANCING (ballet, breakdance, standard, Latin ...)
- WATER SPORTS (canoeing, diving, rowing, swimming, sailing, diving ...)
- WINTER SPORTS (biathlon, bobsleigh, curling, ice hockey, figure skating, skiing, snowboarding ...)
- TREND SPORTS & other (golf, modern pentathlon, skateboarding, skating, sport climbing, surfing, triathlon ...)

**Please enter your gender:**

- Male
- Female
- Divers

**Please enter your age in years:**

(please state this in numbers)

_________ years

**Please enter your height in centimetres:**

(please state this in numbers)

_________ cm

**Please enter your current weight in kilograms:**

_________ kg

**Please state your current marital status:**

- Single
- Married
- In relationship
- Divorced/ seperated
- Widowed
- Other

**Please enter your highest educational level:**

- University degree
- Highschool diploma
- Vocational training
- Secondary school certificate
- Still in school education
- No degree
- Other

**How would you describe your current financial situation?**

Please use the slider to give your answer.

0 = There is not enough in front and behind

10 = I don't need to restrict myself in any way

0 1 2 3 4 5 6 7 8 9 10

**Have you ever had a sports injury?**

**If yes, how often have you had a sports injury in the last 12 months?**

In principle, any injury that occurs during sporting activities is a sports injury. In this case, high forces applied directly or indirectly lead to an acute overload of the tissue's resilience limits and thus to its destruction. From an aetiological point of view, the term "sports injury" includes not only external forces (forces acting on the body from outside) but also internal forces (forces arising within the body itself). [Lohrer H., 2003]

|  | **Never** | **1-2x  per year** | **3-5x  per year** | **6-20x  per year** | **More than 20x per year** |
| --- | --- | --- | --- | --- | --- |
| minor injuries |  |  |  |  |  |
| moderate  injuries |  |  |  |  |  |
| serious  injuries |  |  |  |  |  |
| surgical intervention necessary |  |  |  |  |  |

**Please indicate how often you consume the following substances:**

|  | **Never** | **Rarely** | **Sometimes** | **Often** | **Daily** |
| --- | --- | --- | --- | --- | --- |
| Cannabis |  |  |  |  |  |
| Nicotine |  |  |  |  |  |
| Sedatives prescribed by physicians (e.g. benzodiazepines) |  |  |  |  |  |
| Painkillers prescribed by physicians (e.g. tramadol) |  |  |  |  |  |
| Sedatives not prescribed by physicians/over-the-counter sedatives |  |  |  |  |  |
| Painkillers not prescribed by physicians/ over-the-counter painkillers (e.g. ibuprofen, diclofenac) |  |  |  |  |  |

**Here are some questions about online psychological support services**

Please read our description of online psychological support services, as this brief explanation is relevant for the rest of the questionnaire.

Psychological online support services are all services that you can access via smartphone, tablet or computer and that serve to reduce psychological stress.

Such services can include relaxation techniques, provide information or train skills.

Such services can be used in addition to psychotherapy or to bridge longer waiting times until psychotherapy begins. They can also be used as an independent support method for everyday life.

**Have you read the definition carefully?**

- No
- Yes

**How long do you use the Internet every day for ...?**

|  | **Never** | **Less than 1 hour** | **1 to 3 hours** | **3 to 5 hours** | **More than 5 hours** | |
| --- | --- | --- | --- | --- | --- | --- |
| … private purposes? |  |  |  |  |  | |
| …professional purposes? |  |  |  |  |  | |
| **How confident are you in dealing with ...?** | | | | | |  |
|  | **Not confident at all** | **Rather uncon-fident** | **Neither nor** | **Rather confident** | **Very confident** | |
| ... digital media? |  |  |  |  |  | |
| ... platforms on the Internet? |  |  |  |  |  | |
| ... digital end devices (e.g. computers)? |  |  |  |  |  | |
| **Please evaluate the following statements:** | | | | | |  |
|  | **Do not agree at all** | **Rather disagree** | **Neither nor** | **Rather agree** | **Agree com-pletely** | |
| I feel burdened by the constant availability via mobile phone or email. |  |  |  |  |  | |
| I feel harassed by unsolicited messages and emails. |  |  |  |  |  | |
| I feel uncomfortable wearing a mobile device all the time. |  |  |  |  |  | |
| I have concerns about using the Internet. |  |  |  |  |  | |
| I fear I might be making an irrevocable mistake when using the internet. |  |  |  |  |  | |
| The internet is something that concerns me. |  |  |  |  |  | |

**The following statements relate to health information on the Internet.**

Please read through the individual statements at your leisure and assess the extent to which you agree with them below.

|  | **Do not agree at all** | **Rather disagree** | **Neither nor** | **Tend to agree** | **Fully agree** |
| --- | --- | --- | --- | --- | --- |
| I know where I can find helpful health information on the Internet. |  |  |  |  |  |
| I know which pages with health information are available on the Internet. |  |  |  |  |  |
| I am able to critically evaluate websites with health information. |  |  |  |  |  |
| I know how to use health information from the Internet in a way that helps me. |  |  |  |  |  |
| I can distinguish between trustworthy and dubious websites with health information. |  |  |  |  |  |
| I know how to use the internet to get answers to my health questions. |  |  |  |  |  |
| I know how to find websites with helpful health information. |  |  |  |  |  |
| I feel confident using information from the internet to make decisions about my health. |  |  |  |  |  |

**The following statements are about how you normally behave.**

Please read the statements and tick how much you agree with each statement.

|  | **Do not agree at all** | **Rather disagree** | **Neither nor** | **Tend to agree** | **Fully agree** |
| --- | --- | --- | --- | --- | --- |
| Sometimes I spontaneously do things that I would have been better off not doing. |  |  |  |  |  |
| To feel better, I sometimes do things that I later regret. |  |  |  |  |  |
| I usually think carefully before I do anything. |  |  |  |  |  |
| I usually make my decisions after careful and logical consideration. |  |  |  |  |  |
| I finish what I have started. |  |  |  |  |  |
| I organise my time well so that I can complete tasks on time. |  |  |  |  |  |
| I am prepared to take risks. |  |  |  |  |  |
| I am willing to take a chance. |  |  |  |  |  |
